# Biodegradation Study of Microcrystalline Chitosan and Microcrystalline Chitosan/β-TCP Complex Composites

**DOI:** 10.3390/ijms13067617

**Published:** 2012-06-21

**Authors:** Luciano Pighinelli, Magdalena Kucharska, Maria Wísniewska-Wrona, Bogdan Gruchała, Kinga Brzoza-Malczewska

**Affiliations:** Institute of Biopolymers and Chemical Fibers—IBWCh, Sklodowskiej-Curie 19/27, Lodz, 90-570, Poland; E-Mails: m.kucharska@ibwch.lodz.pl (M.K.); majka.wrona@op.pl (M.W.-W.); biomater@ibwch.lodz.pl (B.G.); brzozak@poczta.onet.pl (K.B.-M.)

**Keywords:** microcrystalline chitosan (MCCh), calcium phosphate, biodegradation, hard tissue regeneration

## Abstract

Bone repair or regeneration is a common and complicated clinical problem in orthopedic surgery. The importance of natural polymers, such as microcrystalline chitosan, and minerals such as HAp and β-TCP, has grown significantly over the last two decades due to their renewable and biodegradable source, increasing the knowledge and functionality of composites in technological and biomedical applications. This study compares the biodegradation process, bioactivity, structure, morphology, and mechanical properties of microcrystalline chitosan and microcrystalline chitosan/β-TCP complex; the latter according to the new method of preparation. The complex showed a homogeneous network structure with regular pores, good bioactivity, even after 60 days of conducting the hydrolytic and enzymatic degradation process, showing a bacteriostatic and bactericidal activity. The complex indicates that it could be used successfully as a base for implants and scaffolds production in orthopedic surgery.

## 1. Introduction

Recent developments in the area of artificial bone materials involve ceramics, which are bio-inert such as alumina and zirconia, resorbable like a tri-calcium phosphate, and bioactive like a hydroxyapatite. Ceramic applications in hard tissue regeneration and replacement are well documented in the literature in the fields of their applications such as replacements of hips, knees, teeth, tendons, and ligaments and repair for periodontal disease, maxillofacial reconstruction, augmentation and stabilization of the jaw bone, spinal fusion, and bone repair after tumor surgery [1–3].

Polysaccharides, such as chitosan and its derivatives like microcrystalline chitosan (MCCh), have some excellent properties for medical applications: non-toxicity (monomer residues are not harmful to health); water solubility or high swelling ability after simple chemical modification; stability to pH variations; biocompatibility; antibacterial, antifungal and antiviral activity; high adhesiveness; and extensive chemical reactivity. Moreover, these materials evidence a strong ability to create hydrogen and ionic bonds and bio-stimulation of natural resistance by controlling and improving bioactivity. These properties make chitosan a good candidate for the preparation and modification of modern generation of scaffolds for tissue regeneration [2,4–8].

Much attention has been given to different materials or composites including organic and inorganic materials that can be used as a base material for scaffold devices, as modification tools for currently used biomedical devices that improve hard and soft tissue regeneration and/or reinforcement efficacy. Additionally, they are applicable as a tissue formation precursor in regenerative therapy in the field of periodontics, orthopedics, cancer, plastic surgery, and veterinary applications [9–17].

The degradation behavior of chitosan and dissolution of calcium phosphates in physiological environmental, plays a crucial role in the long-term performance of tissue engineered cell/material construction. The degradation kinetics may affect many cellular processes, including cell growth, tissue regeneration, and host response, the polymer in the case of microcrystalline chitosan also stimulates the forming of monocytes and inhibits the growth of bacteria and fungi thus lowering the risk of wound inflammation. Some characteristics of the scaffold material such as porosity, particle size and particle shape were reported to have a significant influence on the inflammatory response and reparative bone formation. Irregularly shaped, sharp-edged particles prompted higher inflammatory response than round particles of the same size [8–10,14–20]. The solution parameters of initial pH, ionic concentration and temperature have a large effect on the rate of scaffold dissolution and even type of calcium phosphate precipitated. Ionic concentration, and therefore pH, will obviously change with time as dissolution progresses and this will in turn affect the dissolution rate. If pH rises above a critical value, cytoxicity will occur [18,21].

This study compares the hydrolytic and enzymatic biodegradation, bioactivity, structure and mechanical properties in sponge form of microcrystalline chitosan and microcrystalline chitosan/β-TCP complex composites, the latter in accordance with earlier investigations of the formulation and methods to prepare a new complex as described in Polish Patent Application in 2010 and 2011 [22–24], which could be potentially useful in the field of bone tissue engineering.

## 2. Results and Discussion

Table 1 shows the composition of samples prepared, named SMC for microcrystalline chitosan, and SMC-TCP for (microcrystalline chitosan/β-TCP complex), to compare the biodegradation, bioactivity, structure and mechanical properties in the form of sponges.

### 2.1. Biodegradation Mass Loss

The assessment of hydrolytic and enzymatic degradation process of the SMC and SMC-TCP in sponge form, was estimated in the course of the degradation: pH, percentage of mass loss of the samples (calculated on the microcrystalline chitosan contained in the biocomposite) and concentration of aminosugars (products of degradation contained in the citric-phosphate buffer). Considering that the preparations only assist in the joining of bones and their role in the organism is short lasting, the testing time was limited to 60 days. The results are shown in Table 2a,b.

Table 2a, shows the hydrolytic degradation process by immersion of the sample into the buffer solution, pH 7.4 at 37 °C. Notice that mass loss of SMC-TCP complex after 60 days, gives evidence that samples are very susceptible to degradation. The mass loss of SMC-TCP complex is slightly above 16% and SMC is around 15% with forming of reductive aminosugars around 1.3% and 2.0% respectively.

The results of enzymatic degradation of biocomposites are estimated by the same parameters and compiled in Table 2b. As can be seen, the biocomposites are also susceptible to enzymatic degradation after 60 days and the intensity depends on the applied lysozyme concentration at 200 μg/cm^3^, the mass loss of the SMC-TCP around 18%, and SMC biocomposite around 20%, with higher concentration of aminosugars as a result of degradation amounting to 7.09% and 10.68% respectively. This result suggests that in the SMC-TCP complex sample, the calcium phosphate (β-TCP) slightly shields the polymer against the lysozyme by crosslinking, and probably ionic bonds between ion phosphate and the amino group of polymer, affecting the time and amount of degradation in the microcrystalline chitosan.

### 2.2. SEM Study of the Composites in Sponge Form

**Figure 1:**
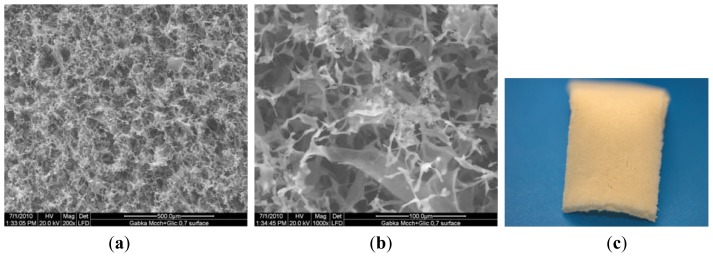
SEM pictures of SMC before degradation process. (**a**) 200×; (**b**) 1000×; (**c**) digital picture.

**Figure 2:**
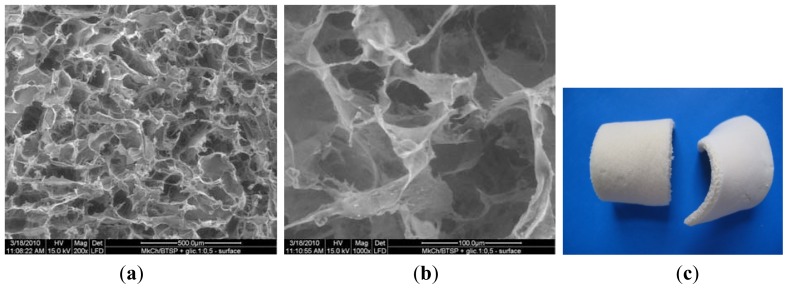
SEM pictures of SMC-TCP complex before degradation process. (**a**) 200×; **(b**) 1000×; (**c**) digital picture.

In this section, the structure and morphology of the samples before and after the biodegradation process are presented according to the preparation conditions listed in Table 1. Figure 1a,b are SEM pictures of SMC before the degradation process, and Figure 2c is the digital picture of sample SMC.

Figure 2a,b are SEM pictures of the SMC-TCP before the degradation process, and Figure 2c is the digital picture of sample SMC-TCP.

**Figure 3:**
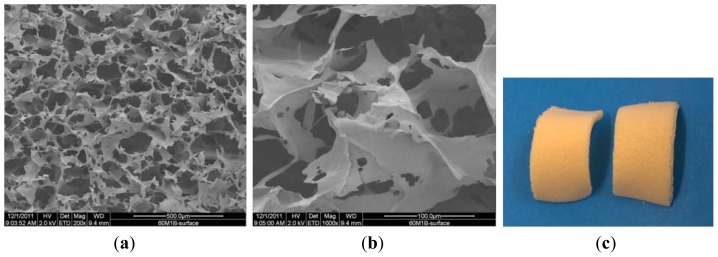
SEM pictures of SMC after 60 days of hydrolytic degradation process. (**a**) 200×; (**b**)1000×; (**c**) digital picture.

Figure 3a,b are SEM pictures of SMC after 60 days of the hydrolytic degradation process, and Figure 3c is a digital picture of sample 60/SMC.

**Figure 4:**
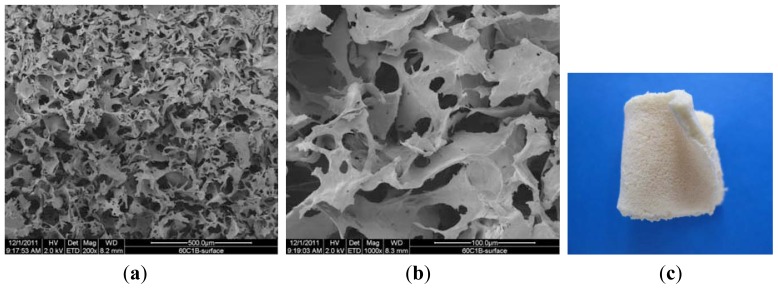
SEM pictures of SMC-TCP complex after 60 days of hydrolytic degradation process. (**a**) 200×; (**b**) 1000×; (**c**) digital picture.

Figure 4a,b are SEM pictures of the of SMC-TCP complex after 60 days of the hydrolytic degradation process, and Figure 4c is a digital picture of sample 60/SMC-TCP.

**Figure 5:**
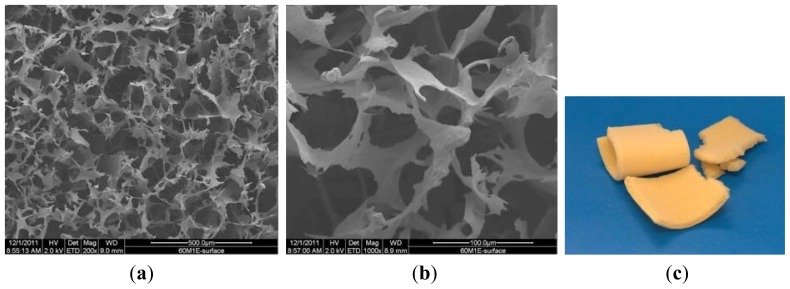
SEM pictures of the SMC after 60 days of enzymatic degradation process. (**a**) 200×; (**b**) 1000×; (**c**) digital picture.

Figure 5a,b are SEM pictures of the SMC after 60 days of the enzymatic degradation (concentration of lysozyme: 200 μg/cm^3^) and Figure 5c is a digital picture of sample 60/SMC.

**Figure 6:**
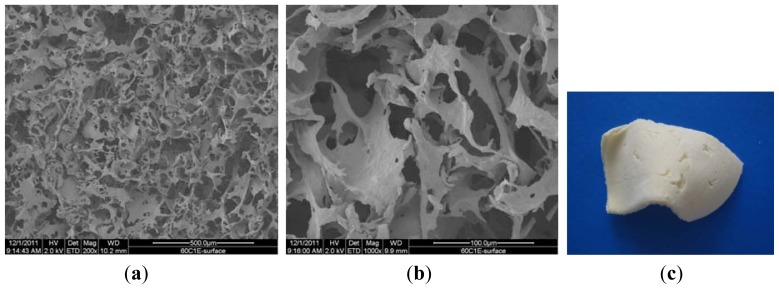
SEM pictures of SMC-TCP complex after 60 days of enzymatic degradation process. (**a**) 200×; (**b**) 1000×; (**c**) digital picture.

Figure 6a,b are SEM pictures of SMC-TCP complex after 60 days of the enzymatic degradation (concentration of lysozyme: 200 μg/cm^3^) and Figure 6c is the digital picture of sample 60/SMC-TCP complex.

The morphology of the microcrystalline chitosan and complex sponges changed progressively with time. In the case of the complex, the sample contained pores that became more compact over time.

Comparing the samples before and after degradation process indicates that under the action of the enzyme at 200 μg/cm^3^ concentration, the surface structure of the sponge undergoes changes with the formation of new and interconnected wider pores. The complex structure reveals a homogenous distribution of micro and nanosized particles and clusters formation of β-TCP in the polymer matrix. The white color of the sample in the digital picture results from the calcium phosphate particles in the complex.

### 2.3. Mechanical Properties of the Sponges

The assessment of mechanical properties of the SMC and the SMC-TCP complex in sponge form are presented in (Table 3a,b) in respect to strength, elongation and elastic modulus, qualities that are crucial for implants in orthopedical surgery. The addition of glycerol interfered with MCCh chains, decreasing intermolecular attraction and increasing polymer chains mobility, maintaining the homogenous porous network structure which facilitates elongation. In the case of the complex with β-TCP, it had little effect on improving the elongation at break; on the other hand a slight decrease in tensile strength, but showing overall a good interaction (ionic and covalent bonding) between the calcium phosphate and the MCCh, in accordance with the literature [22].

After biocomposite materials were affected by 60 days of enzymatic and hydrolytic degradation, a similar behavior of mechanical properties was observed, with lower values of tensile strength, elastic modulus and with higher elongation at break due to the process. The SMC-TCP preparation with best mechanical parameters justifies further investigations. In the case of enzymatic and hydrolytic degradation, a similar behavior was observed, with lower values of tensile strength, elastic modulus with higher elongation at break due to the process.

### 2.4. Bioactivity

The results of the bioactivity investigations are presented in (Table 4a,b), the following test was estimated in the course of antibacterial activity (bacteriostatic and bactericidal activity) using a quantitative test according to standard JIS L 1902:2002 with *Escherichia coli* and *Staphylococcus aureus*.

Both samples SMC and the SMC-TCP complex showed a bacteriostatic activity and a bactericidal activity against *Escherichia coli*; the SMC showing higher activity than the SMC-TCP complex, suggesting more free amino groups from SMC and lower free amino groups from the complex, that are probably bonded with the phosphate groups of the calcium phosphate, reducing the activity.

In the case of *Staphylococcus aureus,* SMC and SMC-TCP showed a bacteriostatic activity and no bactericidal activity was found.

## 3. Experimental Section

The following materials were used:

Microcrystalline chitosan (MCCh paste)-average molecular weight (M_w_) = 330 kD, deacetylation degree (DD) = 82%, ash content = 0.7%, water retention value (WRV) = 598%, polymer content = 2.79%, pH = 7.38.

MCCh/β-TCP complex paste-water retention value (WRV) = 560%, complex content = 3.76%, content of MCCh: 3.008% and β-TCP: 0.752%, pH = 7.40.

Tri-calcium orthophosphate (β-TCP) powder, (Ca_3_(PO_4_)_2_)—Sigma-Aldrich Lab., Munich, Germany, with 425,400 ppm Ca and 199,000 ppm P.

Hydroxyapatite powder (Ca_10_(PO_4_)_6_(OH)_2_—Sigma-Aldrich Lab., Munich, Germany, with 447,200 ppm Ca and 186,500 ppm P.

Plasticiser-Glycerol (C_3_H_8_O_3_), 99%, pure p.a., Sigma-Aldrich, Munich, Germany. Phosphate buffer–pH = 7.40

Lysozyme–muramidase from chicken protein, EC 3.2.1.17, by Merck Co. with an activity of 50,000 U/mg.

### 3.1. Preparation of the MCCh/β-TCP Complex

The MCCh/β-TCP complex was prepared according to Poland Patent Application P 393758 [22].

### 3.2. Preparation of the Composites in Sponge Form

Sponges were prepared via a freeze-drying method using an ALFA 1−4 dryer made by Christ Co. in the temperature range from −25 to 10 °C with a vacuum ranging from 0.1 to 0.53 mbar for 20 to 24 h, depending upon the size of the charge. Freeze-drying resulted in the preparation of sponges with a smooth surface that was free of defects. The compositions of the samples (MCCh and the MCCh/β-TCP complex sponges) are shown in Table 1.

### 3.3. SEM Study of the Composites in Sponge Form

The pores morphology, their size and distribution in the polymer sponge were observed using a scanning electron microscope (SEM, FEI Quanta 200, Oregon, USA).

### 3.4. Mechanical Properties

The mechanical properties of the composites in sponge form were determined in the Accredited Laboratory of Metrology at Institute of Biopolymers and Chemical Fibers (IBWCh) (certificate No. AB 388) using a dynamometer Instron (type 5544) in accordance with the following standards: Polish Standard PN-EN-ISO 527-1, Polish Standard PN-EN-ISO 527-3, and Polish Standard PN-ISO 4593.

### 3.5. Assessment of the Degradability of the Composites

Tested composites were put into a phosphate buffer at pH = 7.41 and bath module 1:300 w/w and sterilised in an autoclave at 121 °C for 25 minutes. Then, samples were dried under vacuum for 15 minutes. The susceptibility of the bio-composites to biodegradation was assessed in the buffer solution with the addition of lysozyme to the amount of 200 μg/cm^3^. The test was conducted in an incubator at 37 °C under static conditions according to standard PN-81C-06504 “preparation of buffer solutions”.

Bio-composites in the form of sponges were removed from bath after 20, 40, and 60 days. Next, the samples were filtered in a Buchner funnel, washed with distilled water at 50 °C, poured over 70% ethanol, filtered after 5 minutes, and dried under vacuum at 70 °C until a constant mass.

The estimation of the hydrolytic and enzymatic degradation progress of the tested preparations was based on the change of the pH, measurements of the concentration of aminosaccharides (products of degradation) in the bath, and the mass loss of the samples.

### 3.6. Bioactivity

A quantity of 0.4 g of tested samples and 0.4 g of reference samples (cotton) were put into separate vessels and sterilized in an autoclave at 121 °C for 25 minutes. After sterilisation and drying, all samples were inoculated with a suspension of bacteria for the determination of antibacterial activity using a quantitative test according to standard JIS L 1902:2002. *Escherichia coli* (ATCC 11229) and *Staphylococcus aureus* (ATCC 6538) were used as testing microorganisms.

## 4. Conclusions

The investigation compared two different microcrystalline chitosans biocomposites designed for bone reconstruction and regeneration.

The biocomposite sponges maintained a homogeneous and interconnected pore network structure after 60 days of biodegradation processes, with a reduction in the size of the pores and the clusters formation in the case of SMC-TCP.

The SMC and SMC-TCP in sponge form are susceptible to hydrolytic and enzymatic degradation processes.

The hydrolytic degradation, by immersing the sample into a buffer solution of pH 7.4 at 37 °C, showed the mass loss of SMC-TCP complex is slightly above 16% and SMC is around 15%, with formation of reductive aminosugars around 1.3% and 2.0% respectively.

With regard to enzymatic degradation in the presence of lysozyme, under the action of the enzyme at a 200 μg/cm^3^ concentration, the mass loss of the SMC-TCP was around 18% and SMC biocomposite around 20%, with a higher concentration of aminosugars as a result of degradation amounting to 7.09% and 10.68% respectively.

The mechanical properties after 60 days of enzymatic and hydrolytic degradation were similar, with lower values of tensile strength, elastic modulus and with higher elongation at break showing that they can be processed in elastic forms enabling the potential to be used as fillers in hard tissue regeneration.

Both SMC and the SMC-TCP complex displayed a bacteriostatic activity and a bactericidal activity against *Escherichia coli*. In the case of *Staphylococcus aureus,* both showed only a bacteriostatic activity.

## Figures and Tables

**Table 1:** Samples composition.

Sample Symbol	Dry Sample Composition * [wt%]
SMC	MCCh: 66.7; Glycerol: 33.3
SMC-TCP	MCCh: 53.4; β-TCP: 13.3; Glycerol: 33.3

* moisture not taken into account; The glycerol was used as a plastisizer for a better manufacture of the samples, increasing the mixing process in the preparation of the suspension and directly related with the homogenous interconnected pore morphology structure, showing smooth surface without defects. Notice the addition of glycerol increases the number and round shape of pores in freeze-dried method in all samples.

**Table 2a:** Percentage mass loss and saccharification before and after hydrolytic degradation.

Time in Days/Sample	Polymer Content in Sample before Degradation [g]	Polymer Content in Sample after Degradation [g]	Mass Loss [%]	Saccharification [%]	pH
0/SMC	0.2273	0.1937	0	0	7.50
0/SMC-TCP	0.3132	0.2842	0	0	7.39
60/SMC	0.2237	0.1885	15.70	1.99	7.47
60/SMC-TCP	0.3373	0.2828	16.15	1.31	7.44

**Table 2b:** Percentage mass loss and saccharification before and after enzymatic degradation.

Time in Days/Sample	Polymer Content in Sample before Degradation [g]	Polymer Content in Sample after Degradation [g]	Mass Loss [%]	Saccharification [%]	pH
0/SMC	0.2273	0.1937	0	0	7.50
0/SMC-TCP	0.3132	0.2842	0	0	7.39
60/SMC	0.1878	0.1507	19.75	10.68	7.47
60/SMC-TCP	0.3159	0.2594	17.90	7.09	7.47

**Table 3a:** Mechanical properties of sponges before and after hydrolytic degradation.

			Parameters		
			
Samples	Degradation Time [days]	Composition	Tensile Strength [MPa]	Elongation at Break [%]	Elastic Modulus [MPa]
SMC	0	MCCh: 66.7 Glycerol: 33.3	0.1720	1.53	1.400
SMC-TCP	0	MCCh: 53.4 β-TCP: 13.3 Glycerol: 33.3	0.0150	2.41	1.000
SMC	60	MCCh: 66.7 Glycerol: 33.3	0.0091	3.19	0.010
SMC-TCP	60	MCCh: 53.4 β-TCP: 13.3 Glycerol: 33.3	0.0148	5.65	0.005

**Table 3b:** Mechanical properties of sponges before and after enzymatic degradation.

			Parameters		
			
Samples	Degradation Time [days]	Composition	Tensile Strength [MPa]	Elongation at Break [%]	Elastic Modulus [MPa]
SMC	0	MCCh: 66.7 Glycerol: 33.3	0.172	1.53	1.40
SMC-TCP	0	MCCh: 53.4 β-TCP: 13.3 Glycerol: 33.3	0.015	2.41	1.00
SMC	60	MCCh: 66.7 Glycerol: 33.3	0.0073	1.88	0.005
SMC-TCP	60	MCCh: 53.4 β-TCP: 13.3 Glycerol: 33.3	0.0055	3.14	0.015

**Table 4a:** Determination of antibacterial activity (*Escherichia coli*).

Sample	Time [h]	Number of Living Bacteria [cfu/sample]	Confidence Interval [cfu/sample]	Bacteriostatic Activity	Bactericidal Activity
Control	0	1.1 × 10^5^	9.1 × 10^4^–1.4 × 10^5^	0	0
Control	24	1.4 × 10^8^	1.2 × 10–1.7 × 10^8^	0	0
SMC	24	6.5 × 10^2^	2.6 × 10^2^–1.3 × 10^3^	5.3	2.2
SMC-TCP	24	6.7 × 10^6^	4.8 × 10^5^–1.4 ×10^7^	1.3	1.8

**Table 4b:** Determination of antibacterial activity (*Staphylococcus aureus*).

Sample	Time [h]	Number of Living Bacteria [cfu/sample]	Confidence Interval [cfu/sample]	Bacteriostatic Activity	Bactericidal Activity
Control	0	2.7 × 10^4^	2.3 × 10^4^–3.1 × 10^4^	0	0
Control	24	7.4 × 10^6^	4.3 × 10^6^–1.1 × 10^7^	0	0
SMC	24	6.8 × 10^5^	4.4 × 10^4^–1.8 × 10^6^	1.1	−1.4
SMC-TCP	24	3.3 × 10^6^	1.2 × 10^6^–5.2 × 10^6^	0.4	−2.1
